# Lysozyme amyloid: evidence for the W64R variant by proteomics in the absence of the wild type protein

**DOI:** 10.1080/13506129.2020.1720637

**Published:** 2020-02-18

**Authors:** Alexandra Moura, Paola Nocerino, Janet A. Gilbertson, Nigel B. Rendell, P. Patrizia Mangione, Guglielmo Verona, Dorota Rowczenio, Julian D. Gillmore, Graham W. Taylor, Vittorio Bellotti, Diana Canetti

**Affiliations:** a Wolfson Drug Discovery Unit and National Amyloidosis Centre, Centre for Amyloidosis and Acute Phase Proteins, Division of Medicine, University College London, London, UK;; b Department of Molecular Medicine, Institute of Biochemistry, University of Pavia, Pavia, Italy

Human lysozyme is a bacteriolytic enzyme synthesised by gastrointestinal (GI) tract macrophages and hepatocytes. It is found in many different tissues and body fluids including the liver, articular cartilage, saliva and plasma [1]. Lysozyme amyloidosis (ALys) is one of the rarest types of systemic amyloidosis [2]. It is a hereditary, autosomal dominant disease that is associated with a single point mutation in the lysozyme gene. To date, nine amyloid point mutations have been reported (http://amyloidosismutations.com/mut-alys.php).

Proteomics represents a valuable tool for diagnosis and is complementary to immunohistochemistry and gene sequencing. The advantage of proteomics is the ability to directly characterise proteins in the amyloid deposits. In certain circumstances, this approach can be limited by the normal requirement to use trypsin as the proteolytic enzyme.

Here, we report a case of a patient carrying the mutation W64R *(*p.W82R) in which we highlight the importance of the combined use of trypsin and Asp-N to identify the amyloid composition. The W64R lysozyme mutation has been originally described by Valleix et al. [3] in 2002. A similar case of W64R lysozyme amyloid has just been reported by Li et al. [4]. Although the genetic sequencing showed the presence of the mutation, the proteomics output confirmed the presence of lysozyme, but details on fibrils composition were not provided.

**Figure 1.**
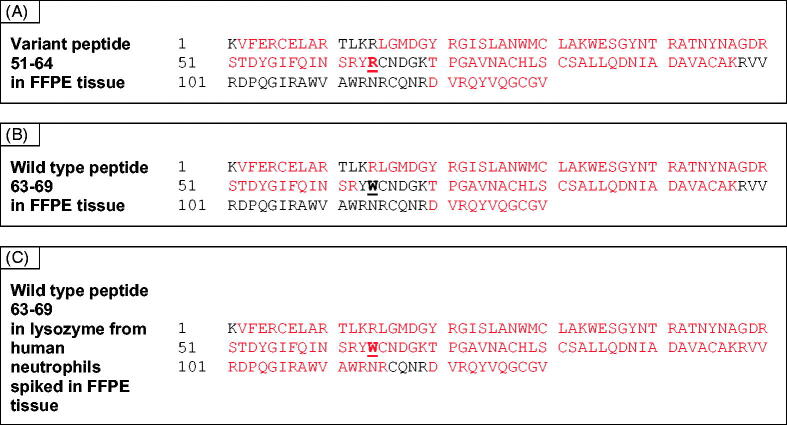
Panel A shows the peptides generated by trypsin treatment of a Congo red positive FFPE tissue from a patient with variant W64R lysozyme. The variant peptide 51–64 is clearly observed. There is no evidence for the equivalent wild type peptide 63–69 (panel B). Panel C shows coverage of wild type lysozyme added to a control FFPE tissue, where the wild type peptide can be observed.

A sample of Congo red positive formalin-fixed paraffin-embedded (FFPE) GI tissue was collected by laser capture dissection and analysed by proteomics using trypsin as the digestion enzyme [5]. Data were then evaluated using MASCOT software to search the Swiss-Prot database to which was appended an additional lysozyme database of nine variants. A number of proteins were present in the sample (Supplementary Table 1) including lysozyme which was identified with high Mascot score (1403) and a protein coverage of 66%. Peptides containing the W64R mutation were not detected since the additional tryptic cleavage site generated by a W→R change resulted in the generation of small fragments outside the normal range of the instrument. A miscleaved variant peptide 51–64, (R)STDYGIFQINSRYR, was observed, however, the confidence in the assignment was low (Figure 1(A) and Supplementary Figure 1(A)). The wild type 63–69 and the miscleaved 63-97 peptides were not detected (Figure 1(B)). To determine whether this was due to the absence of wild type protein in the fibrils, a sample of wild type lysozyme from human neutrophils (Sigma-Aldrich, St. Louis, MO) was spiked into a Congo red negative FFPE GI tissue and analysed by proteomics. Lysozyme was identified with a sequence coverage of 84% and the wild type peptide 63–69 was detected (Figure 1(C)). This is in contrast with that observed in the natural amyloid fibrils of lysozyme where the wild type peptide was absent. Fifteen other proteins were identified in the sample (Supplementary Table 2).

**Figure 2.**
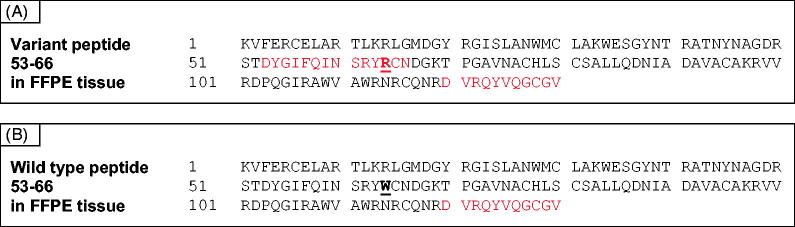
Protein coverage of AspN-digested amyloid. Cleavage by AspN occurs between 52 T and 53 D. The resulting variant peptide 53–66 is shown in red (panel A), the wild type peptide is not covered (panel B).

Proteomic analysis following Asp-N digestion was then performed to confirm this finding in a separate specimen of Congo red positive GI amyloid. Lysozyme was identified together with a number of other proteins (Supplementary Table 3). Lysozyme protein coverage was only 7% obtained by Asp-N digestion and this is much more limited compared to the coverage achieved by trypsin digestion. However, the peptide 53–66, DYGIFQINSRYRCN(D), containing the tryptophan to arginine amino acid substitution, was identified after Asp-N digestion (Figure 2(A) and Supplementary Figure 1(B)). There was no evidence for the wild type peptide (Figure 2(B)).

In conclusion, we have confirmed the original observation that the wild type lysozyme cannot be detected in natural amyloid fibrils [2]. This is consistent with the resistance of wild type lysozyme to the amyloid conversion even in the presence of natural seeds of fibrils of the lysozyme variant, making ALys different from other types of amyloidosis such as that caused by transthyretin mutations.

Further, we stress the importance of the combined use of trypsin and other enzymes such as Asp-N to specifically detect the variant peptide in the amyloid tissue.

Alexandra Moura, Paola Nocerino, Janet A. Gilbertson and Nigel B. Rendell *Wolfson Drug Discovery Unit and National Amyloidosis Centre, Centre for Amyloidosis and Acute Phase Proteins, Division of Medicine, University College London, London, UK* P. Patrizia Mangione *Wolfson Drug Discovery Unit and National Amyloidosis Centre, Centre for Amyloidosis and Acute Phase Proteins, Division of Medicine, University College London, London, UK* *Department of Molecular Medicine, Institute of Biochemistry, University of Pavia, Pavia, Italy* Guglielmo Verona, Dorota Rowczenio, Julian D. Gillmore and Graham W. Taylor *Wolfson Drug Discovery Unit and National Amyloidosis Centre, Centre for Amyloidosis and Acute Phase Proteins, Division of Medicine, University College London, London, UK* Vittorio Bellotti *Wolfson Drug Discovery Unit and National Amyloidosis Centre, Centre for Amyloidosis and Acute Phase Proteins, Division of Medicine, University College London, London, UK* *Department of Molecular Medicine, Institute of Biochemistry, University of Pavia, Pavia, Italy* Diana Canetti *Wolfson Drug Discovery Unit and National Amyloidosis Centre, Centre for Amyloidosis and Acute Phase Proteins, Division of Medicine, University College London, London, UK* d.canetti@ucl.ac.uk
